# Computational Study on the Different Ligands Induced Conformation Change of β2 Adrenergic Receptor-Gs Protein Complex

**DOI:** 10.1371/journal.pone.0068138

**Published:** 2013-07-29

**Authors:** Qifeng Bai, Yang Zhang, Yihe Ban, Huanxiang Liu, Xiaojun Yao

**Affiliations:** 1 College of Chemistry and Chemical Engineering, Lanzhou University, Lanzhou, China; 2 School of Information Science and Engineering, Lanzhou University, Lanzhou, China; 3 School of Pharmacy, Lanzhou University, Lanzhou, China; 4 Key Lab of Preclinical Study for New Drugs of Gansu Province, Lanzhou University, Lanzhou, China; Wake Forest University, United States of America

## Abstract

β_2_ adrenergic receptor (β_2_AR) regulated many key physiological processes by activation of a heterotrimeric GTP binding protein (Gs protein). This process could be modulated by different types of ligands. But the details about this modulation process were still not depicted. Here, we performed molecular dynamics (MD) simulations on the structures of β_2_AR-Gs protein in complex with different types of ligands. The simulation results demonstrated that the agonist BI-167107 could form hydrogen bonds with Ser203^5.42^, Ser207^5.46^ and Asn293^6.55^ more than the inverse agonist ICI 118,551. The different binding modes of ligands further affected the conformation of β_2_AR. The energy landscape profiled the energy contour map of the stable and dissociated conformation of Gαs and Gβγ when different types of ligands bound to β_2_AR. It also showed the minimum energy pathway about the conformational change of Gαs and Gβγ along the reaction coordinates. By using interactive essential dynamics analysis, we found that Gαs and Gβγ domain of Gs protein had the tendency to separate when the inverse agonist ICI 118,551 bound to β_2_AR. The α5-helix had a relatively quick movement with respect to transmembrane segments of β_2_AR when the inverse agonist ICI 118,551 bound to β_2_AR. Besides, the analysis of the centroid distance of Gαs and Gβγ showed that the Gαs was separated from Gβγ during the MD simulations. Our results not only could provide details about the different types of ligands that induced conformational change of β_2_AR and Gs protein, but also supplied more information for different efficacies of drug design of β_2_AR.

## Introduction

The β_2_ adrenergic receptor (β_2_AR) belonged to class A G protein-coupled receptors (GPCRs) [1] and regulated many key physiologically processes such as smooth muscle relaxation in the airways and the vasculature [2]–[7]. During the past years, much progress had been made in the determination of the crystal structure of β_2_AR with different types of ligands. The crystal structure of β_2_AR in complex with the inverse agonist carazolol was determined in 2007. It revealed the inactive conformation of β_2_AR [8]. The neutral antagonist alprenolol bound to β_2_AR structure was reported in 2010. This work showed that the antagonist could block agonist signal but maintain basal signal [9]. The irreversible agonist-β_2_AR complex was reported in 2011. This agonist was irreversible because it was covalently tethered to a specific site of β_2_AR [10]. At the same time, a reversible agonist-β_2_AR in complex with the camelid antibody fragment that exhibited G protein-like behavior was obtained by X-ray crystallography [11]. Besides, Rasmussen *et al*. reported the crystal structure of agonist-occupied β_2_AR and nucleotide-free Gs heterotrimer (α, β and γ). This work gave a model system for understanding the detailed mechanism about the activation of Gs and also for understanding the ligands induced conformation change of β_2_ adrenergic receptor-Gs (β_2_AR-Gs) protein complex [12]. The analysis of β_2_AR-Gs complex could provide some information about the essential mechanism of structural events linking GPCR-Gs protein complex formation by using peptide amide hydrogen-deuterium exchange mass spectrometry [13]. Engineering and characterization of β_2_AR-based on ion-channel coupled receptors gave new insights into the conformational dynamics of β_2_AR [14]. All these studies also indicated that it was difficult to obtain the crystal structure of the agonist-bound to active conformation of β_2_AR if the G protein did not bind to β_2_AR.

Even though the active conformation of β_2_AR-Gs have been resolved, it was still difficult to obtain the detailed information about the dynamic process of inactive or active state of β_2_AR-Gs from real experiments. Compared with experimental study, all atoms molecular dynamics simulations [15]–[20] and coarse-grained molecular dynamics simulations [21], [22] methods could provide much more dynamic information at the atomic level about the activation or inactivation mechanism of β_2_AR. Other computational methods such as molecular docking and conformational analysis [23]–[27] were also successfully used to study the function and activation mechanism as well as to discovery the small molecular ligands of β_2_AR on basis of the crystal structures. The MD simulations of agonist-β_2_AR complex showed that agonist, inverse agonist and antagonist had different interaction modes with the active sites of β_2_AR. The main reason was that the waters in the cavity of β_2_AR had different contribution to the stabilization of the interaction network [20]. The atomic level description illuminated that drug must cross two energetic barriers to get into the active site of β_2_AR. The first barrier was mainly due to hydrophobic interaction. The second energetic barrier was due to dehydration and allosteric receptor when the drug moved into the binding pocket [28]. In addition, Dror *et al*. proposed that the agonist-β_2_AR could transform momentarily from active to the inactive conformation based on the results of MD simulations. This study also showed β_2_AR had an intermediate state. The conformation of β_2_AR would be induced to active or inactive state if agonist or inverse agonist bound to the cavity of receptor [29]. Provasi *et al*. performed free energy calculation on the crystal structure of β_2_AR with different ligands (either inverse agonists, neural antagonists, or agonists). The simulation results suggested that different type ligands had different free energy landscape. Especially, the agonist had opposite energy barrier to the inverse agonist. And there was nearly no energy barrier when β_2_AR had no ligands in the cavity [30]. Goetz *et al*. studied the interaction between C-terminal end of Gαs and β_2_AR by performing MD simulations [31]. Feng *et al*. carried out 20 ns MD simulations on agonist-bound part of β_2_AR without Gβγ domain to investigate the activation mechanism of β_2_AR [32].

Despite these recent remarkable advances in β_2_AR structure determination and molecular dynamics simulation, the detailed mechanism by which different types of ligands induced dynamic conformational changes of β_2_AR and Gs protein during the modulated process was still not reported. Most of the reported works mainly focused on the complex of β_2_AR and ligands. In order to understand the modulation of Gs by β_2_AR, it was more reliable to perform MD simulation based on the crystal structure of β_2_AR-Gs complex. The following important questions still need to be answered, such as: what is the difference of binding mode between β_2_AR and different kinds of ligands? which kind of ligand could induce Gαs to separate from Gβγ? How did the inactive conformation of β_2_AR interact with Gs protein?

In order to further explore how different types of ligands affected the behavior of Gαs and Gβγ in the β_2_AR-Gs complex. We performed a total of 800 ns MD simulations on the complex of β_2_AR-Gs bound to agonist (BI-167107), antagonist (alprenolol), inverse agonist (ICI 118,551) and their unliganded form with explicit solvent and lipids at constant pressure and constant temperature. The graphics processing unit (GPU) computer was used to accelerate the MD simulations. The analysis of energy landscape was performed to illustrate the minimum energy pathway of the conformational change of Gαs and Gβγ along the reaction coordinates when ICI 118,551 bound to β_2_AR. Furthermore, we used interactive essential dynamics (IED) [33] to identify the dissociation of Gαs and Gβγ by analyzing the MD simulated trajectory. Our simulated results showed that Gαs was separated from the Gβγ when the ICI 118,551 bound to active sites of β_2_AR. Besides, the α5-helix had fast motion relative to TM3, TM5, TM6, TM7 of β_2_AR if the ICI 118,551 bound to β_2_AR. Our results could also provide the information about the inactivation and activation mechanism of Gs protein induced by different types of ligands.

## Results and Discussion

### Structure of β_2_AR-Gs Complex

**Figure 1:**
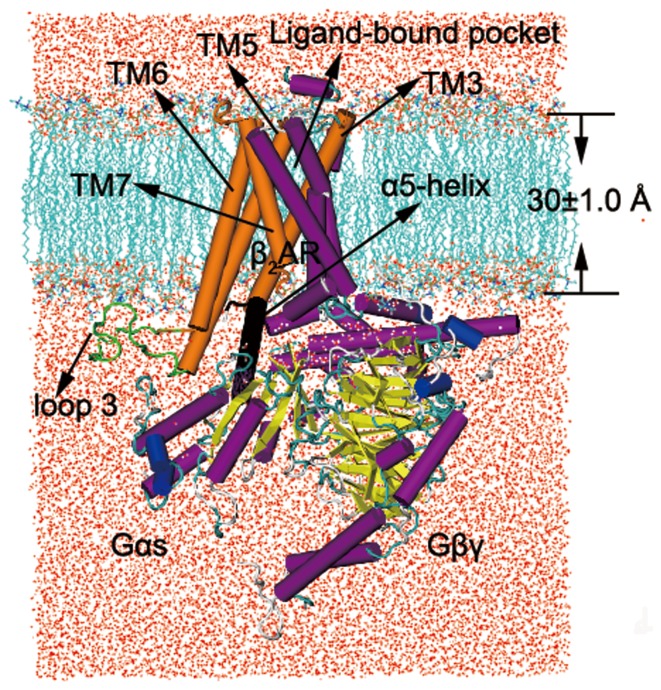
The structure of simulated complex. The red points are water. The cyan lipids represent membrane. The membrane and water only show the positive part of y axis.

**Figure 2:**
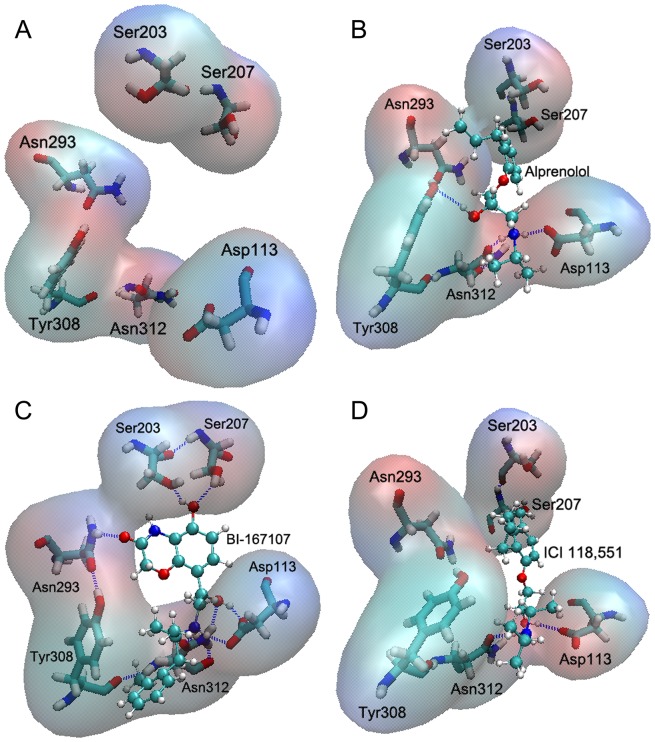
Snapshot of the hydrogen bonds between different ligands and β_2_AR. (A) The binding sites of β_2_AR. (B) Alprenolol forms three hydrogen bonds with Asp113, Tyr308 and Asn312. (C) BI-167107 has five hydrogen bonds with Asp113, Ser203, Ser207, Asn293 and Asn312. (D) ICI 118,551 forms two hydrogen bonds with Asp113 and Asn312.

The structure of β_2_AR-Gs with explicit waters and lipids was shown as in Figure 1. The thickness for membrane location was about 30±1.0 Å, which was calculated by OPM database [34]. The main part of β_2_AR-Gs consisted of β_2_AR, Gαs and Gβγ. The loop between TM5 and TM6 was modeled on basis of the crystal structure of β_2_AR-Gs. TM3, TM5, TM6 and TM7 (TM3,5,6,7) were shown in the origin part of β_2_AR-Gs. The black part was α5-helix. The residues of the active site in the pocket of β_2_AR include Asp113^3.32^, Ser203^5.42^, Ser207^5.46^, Asn293^6.5^, Tyr308^7.35^ and Asn312^7.39^ (see Figure 2A). The space surrounded by these sites was the volume of β_2_AR. The crystal structure of β_2_AR-Gs in complex with the agonist (BI-167107) was used in our simulations In order to get β_2_AR-Gs in complex with different kinds of ligands, the inverse agonist (ICI 118,551) and antagonist (alprenolol) were docked into the pocket of β_2_AR-Gs. The 200 ns MD simulations were performed for β_2_AR-Gs in complex with different ligands on a workstation equipped with four pieces of graphics processing unit (GPU) and two processors with six cores (see Figure S1).

### Ligands Bound to Different Sites of β_2_AR

**Figure 3:**
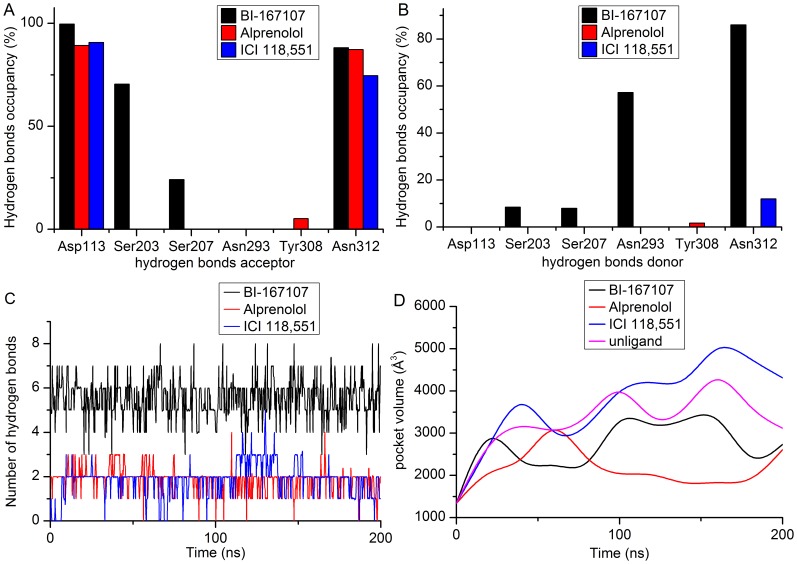
The hydrogen bonds occupancy and volume of binding pocket. (A–B) The column represents the percent of hydrogen bonds occupancy when the residues are as hydrogen bonds acceptor or donor in the pocket of β_2_AR. (C) The total number of hydrogen bonds versus the simulated time. (D) The ligands-bound pocket volume of β_2_AR versus the simulation time.

**Figure 4:**
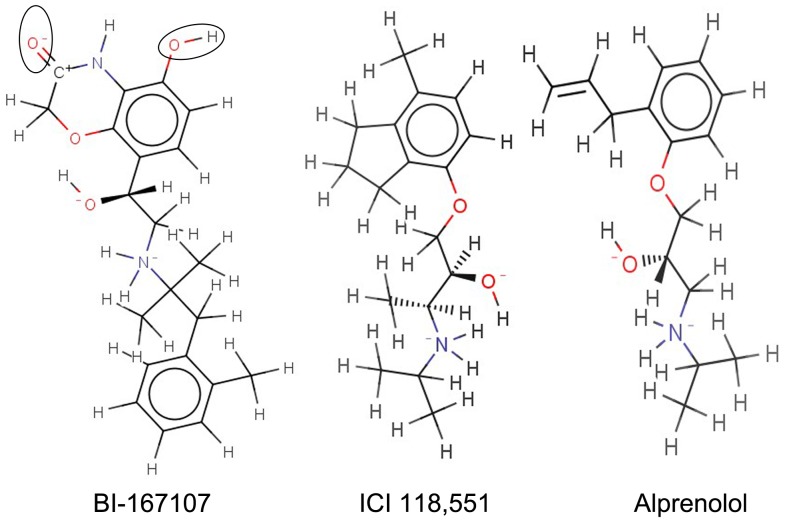
Structures of BI-167107, ICI 118,551 and alprenolol. The oxygen and hydroxyl groups in the black oval form another three hydrogen bonds with the active sites of β_2_AR.

After 200 ns MD simulations, the analysis of hydrogen bonds occupancy showed that inverse agonist (ICI 118,551), antagonist (alprenolol) and agonist (BI-167107) could form hydrogen bonds with different sites of β_2_AR-Gs (Figure 3A and 3B). We also obtained the hydrogen bond interaction between β_2_AR and different ligands (see Figure 2B, 2C and 2D) from the MD simulation trajectory at the same time. ICI 118,551 only had two stable hydrogen bonds with Asp113^3.32^ and Asn312^7.39^ (Figure 2D and Figure 3A). In comparison, BI-167107 had another three stable hydrogen bonds with Ser203^5.42^, Ser207^5.46^ and Asn293^6.55^ besides Asp113^3.32^ and Asn312^7.39^ (Figure 3A, 3B and Figure 2C). Alprenolol had a similar binding mode with ICI 118,551 except lower hydrogen bonds occupancy on Tyr308^7.35^ (Figure 3A, 3B and Figure 2B). The number of hydrogen bonds also showed BI-167107 could form more hydrogen bonds than alprenolol and ICI 118,551 along the simulation time (Figure 3C). The main reason was that BI-167107 had more oxygen and hydroxyl groups than Alprenolol and ICI 118,551 as shown in the black oval of Figure 4, so BI-167107 could be easy to form another three hydrogen bonds with Ser203^5.42^, Ser207^5.46^ and Asn293^6.55^ (see Figure 2C). The results showed that inverse agonist had different binding modes with agonist and antagonist.

In order to measure the pocket change of β_2_AR during the simulations, the pocket detection plugin of VMD [35], [36] was used to calculate the lignad-bound pocket volume versus simulation time (Figure 1 and Figure 3D). The value of the pocket volume of unliganded complex showed that this conformation of β_2_AR was in the intermediate state. The pocket volume would become larger when the inverse agonist ICI 118,551 bound to the pocket of β_2_AR, while the pocket volume would shrink when the agonist BI-167107 or antagonist alprenolol bound to β_2_AR. These results indicated different ligands could adjust the pocket space size of the β_2_AR though different binding modes of β_2_AR. The changes of pocket volume size would further affect the conformation of β_2_AR.

### Conformation CHANGE of β_2_AR Induced by Different Ligands

**Figure 5:**
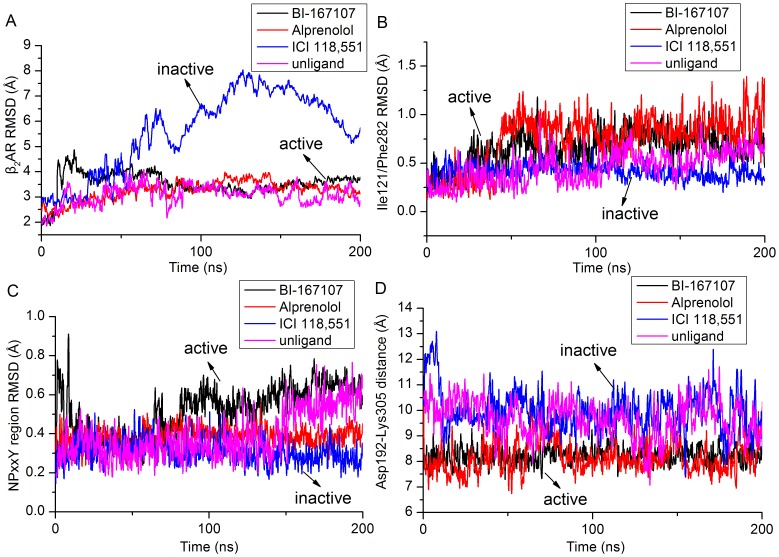
Active and inactive state of β_2_AR. (A) RMSD of the backbone atoms of β_2_AR versus simulation time. (B) Time evolution of RMSD of non-hydrogen atoms of Ile121^3.40^ and Phe282^6.44^. (C) Time evolution of RMSD of the backbone atoms of NPxxY region. (D) Distance of Cα carbons of Asp192^5.31^ and Lys305^7.32^ versus simulation time.

In order to study conformational change of β_2_AR induced by different ligands, the root mean square deviation (RMSD) of the backbone atoms of β_2_AR was measured versus simulation time (Figure 5A). The β_2_AR in complex with ICI 118,551 reached equilibrium phase after 5 ns MD simulations (see Figure S2). The RMSD of β_2_AR-ICI 118,551 still maintained about 2.7 Å until 26 ns MD simulations (Figure 5A). By comparison with the RMSD of β_2_AR-BI-167107, we could see that β_2_AR-ICI 118,551 was still in active conformation. After 26 ns, the conformation of β_2_AR was changed into another state. In order to make sure the conformational feature of β_2_AR, FATCAT rigid algorithm [37] was used to calculate the RMSD with respect to the crystal structure of inverse agonist ICI 118,551-bound β_2_AR (PDB code: 3NY8) (see Table S1). The RMSD values in the Table S1 indicated the simulated conformation was closer to the inactive conformation, while the increased value of RMSD after about 26 ns suggested that simulated structures had different conformation with the agonist-bound β_2_AR (see Figure 5A). The β_2_AR-alprenolol and unliganded form of β_2_AR had similar RMSD with β_2_AR-BI-167107. It suggested that β_2_AR did not change its active state if alprenolol, BI-167107 or no ligand bound to β_2_AR. The active and inactive state of β_2_AR could be identified by some reported sites (Ile121^3.40^/Phe282^6.44^, NPxxY region: Asn322^7.49^-Tyr326^7.53^ and Asp192^5.31^/Lys305^7.32^) [9], [29]. These sites could be used to distinguish the active and inactive conformation of β_2_AR.

Figure 5B illustrated different RMSD of non-hydrogen atoms of Ile121^3.40^/Phe282^6.44^ when ICI 118,551, alprenolol, BI-167107 or no ligand bound to β_2_AR. With the increased time of MD simulations, RMSD of Ile121^3.40^/Phe282^6.44^ of β_2_AR in complex without ligand was up to the same level of agonist, antagonist-bound β_2_AR as shown in Figure 5B. These states represented the active conformation of β_2_AR. In comparison, the lower RMSD of Ile121^3.40^/Phe282^6.44^ of β_2_AR-ICI 118,551 represented the inactive conformation of β_2_AR.

Figure 5C showed the RMSD of the backbone atoms of NPxxY motif which could distinguish different states of β_2_AR. The RMSD of NPxxY region of β_2_AR-unligand was close to the level of β_2_AR-BI-167107 after about 148 ns MD simulations (see Figure 5C). The data also showed that β_2_AR-alprenolol had different RMSD of NPxxY region with unliganded, BI-167107 and ICI 118,551-bound β_2_AR. The possible reason was that the conserved NPxxY region could discern diverse conformations of β_2_AR when different types of ligands bound to β_2_AR.

Figure 5D described the distance of Cα carbons of Asp192^5.31^ and Lys305^7.32^ versus MD simulation time. The distance divided the conformation of β_2_AR into the inactive part and active part because Asp192^5.31^ and Lys305^7.32^ only represented part of extracellular surface of β_2_AR. ICI 118,551 and unligand belonged to inactive part while alprenolol and BI-167107 played an active role.

All these results corresponded to distinct functional behavior of different types of ligands. The inverse agonist ICI 118,551 could block the activating signaling. In contrast, unliganded and alprenolol-bound β_2_AR could maintain the basal activity signaling. BI-167107 could enhance the active signaling of β_2_AR [9].

### Energy Landscape of Gαs and Gβγ

The above simulated results showed that different types of ligands could regulate the diverse states of β_2_AR. Besides, Gαs and Gβγ had shown some interesting conformations when BI-167107, alprenolol, ICI 118,551 or no ligand bound to the active sites of β_2_AR. Our molecular dynamics simulations trajectory of β_2_AR-Gs contained a wide range of conformational spaces. Therefore, abundant information was supplied for the energy landscape analysis of the conformations of Gαs and Gβγ. Two major motions represented the conformations of Gαs and Gβγ: one was the centroid distance of Gαs and Gβγ, the other was the RMSD of Gαs and Gβγ.

**Figure 6:**
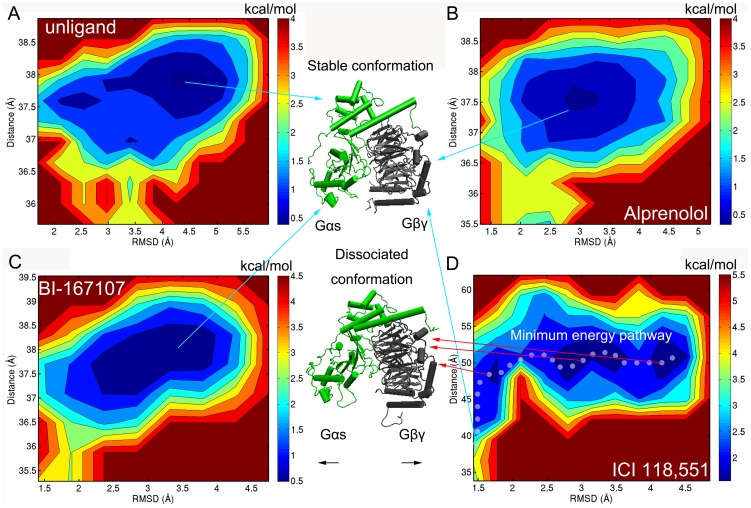
Energy landscape of Gαs and Gβγ. (A–D) The energy landscape map of Gαs and Gβγ in complex without ligand or with alprenolol, BI-167107 and ICI 118,551. Reaction coordinates are defined two parts: the centroid distance between Gαs and Gβγ; the RMSD of Gαs and Gβγ. The white points represent the minimum energy pathway.

Figure 6 illustrated the energy landscape of Gαs and Gβγ corresponding to two reaction coordinates. This energy landscape contained one major deep well when the BI-167107, alprenolol or no ligand bound to β_2_AR (see Figure 6A, 6B and 6C). This energy part represented the stable structure of Gαs and Gβγ which was not separated from each other. However, the energy landscape consisted of three main deep wells when the ICI 118,551 combined with β_2_AR. The white points depicted the minimum energy pathway. It was mainly relevant to the stable conformation of Gαs and Gβγ (0∼43 ns) before the first deep well. Along with the change of simulated time, the Gαs and Gβγ complex passed over an energy barrier of ∼2.0 kcal/mol. At the same time, the stable conformation of Gαs and Gβγ became to dissociated state. It only need overcome the energy barrier of ∼0.5 kcal/mol for each neighboring deep well. These three deep wells represented the dissociated conformation of Gαs and Gβγ (see Figure 6D). In additions, Figure 6D showed the lowest energy barrier of ∼1.5 kcal/mol in the deep well, while Figure 6A, 6B, 6C showed the lowest energy barrier of deep well was ∼0.5 kcal/mol. It further indicated the domain of Gαs and Gβγ was not stable when ICI 118,551 bound to β_2_AR.

### Gαs is Separated from Gβγ

**Figure 7:**
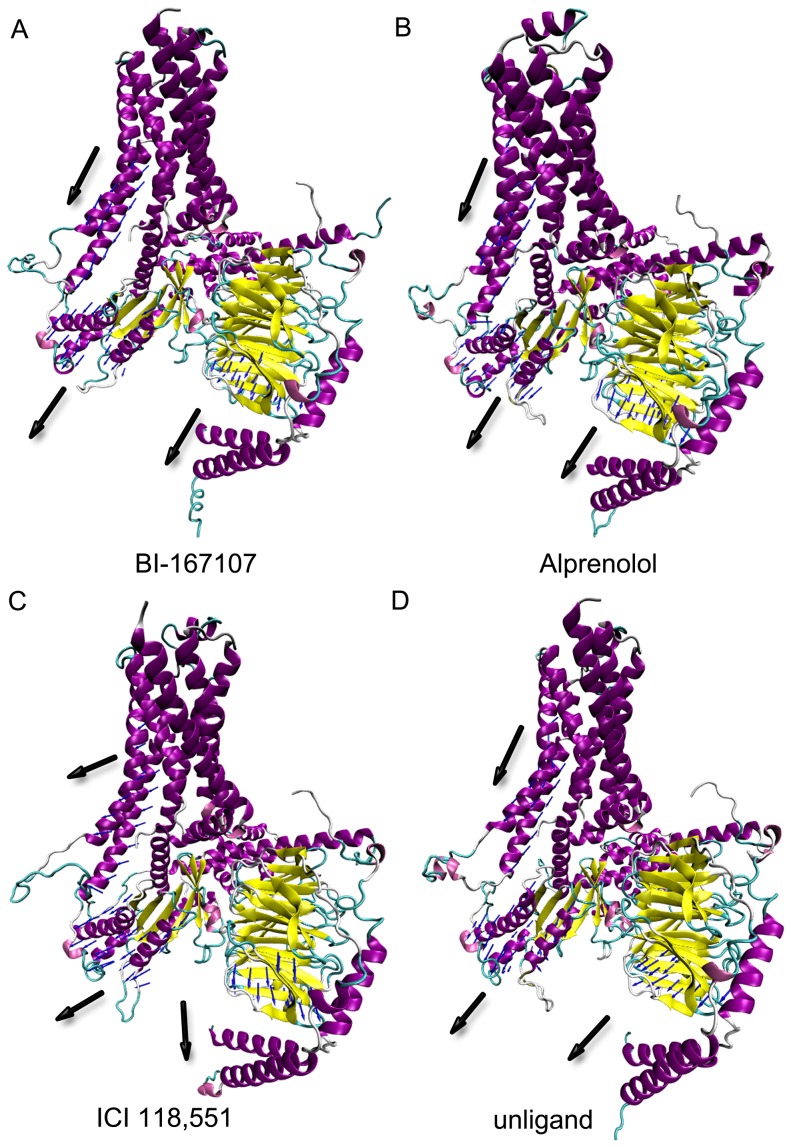
IED plot of principal motions of Gαs and Gβγ. (A–D) Unliganded, BI-167107 and alprenolol-bound β_2_AR has similar movement. Gαs and Gβγ keep the similar direction of motions. ICI 118,551 induces Gαs and Gβγ to separate from each other.

After analysis of the energy landscape of Gαs and Gβγ, it is interesting to study the movement of Gαs and Gβγ. The motions of Gαs and Gβγ were analyzed by interactive essential dynamics (IED) analysis [33]. The two principal components of motions revealed the movements of TM5, TM6 and Gαs and Gβγ (Figure 7). The Gαs did not move away from Gβγ when BI-167107 and alprenolol bound to β_2_AR (Figure 7A and 7B). The Gαs and Gβγ domain was also not dissociated when there was no ligand on the β_2_AR (Figure 7D). In this case, TM5 and TM6 had almost no relative motion. In comparison, the Gαs domain was separated from Gβγ domain when ICI 118,551 bound to β_2_AR. At the same time, TM5 and TM6 had the open tendency with respect to Gβγ domain (Figure 7C).

**Figure 8:**
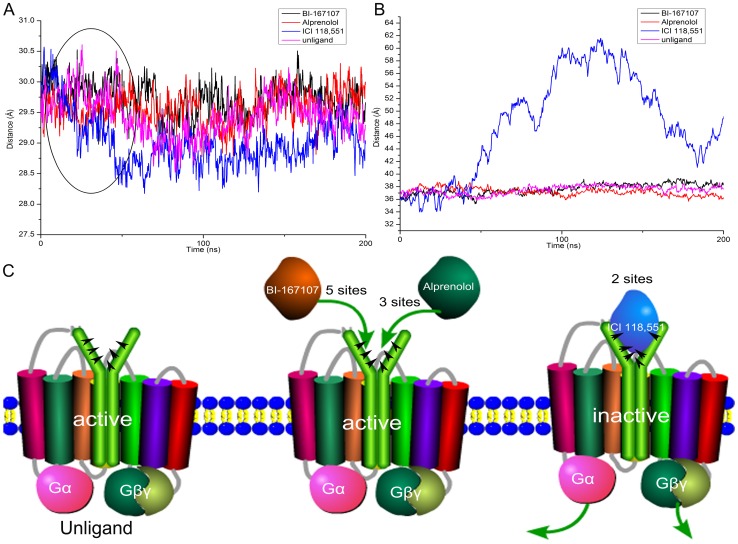
Motions of Gαs and Gβγ domain. (A) The centroid distance of α5-helix and TM3, TM5, TM6, TM7 versus simulation time. (B) Time evolution of centroid distance of Gαs and Gβγ. (C) The cartoon representation of the dissociation mechanism of Gαs and Gβγ.

The α5-helix had been reported to play an important role on the interaction between β_2_AR and Gs protein [12], [13], [32]. The sketch of the structure of α5-helix and TM3,5,6,7 was shown in Figure 1. The centroid distance between α5-helix and TM3,5,6,7 was measured over the simulation time. As shown in black oval of Figure 8A, the centroid distance between α5-helix and TM3,5,6,7 was dropped sharply when ICI 118,551 bound to the pocket of β_2_AR. It indicated that α5-helix moved quickly relative to TM3,5,6,7. After about 43 ns MD simulations, the centroid distance became longer when BI-167107, alprenolol or no ligands was in the active pocket of β_2_AR, while the distance was shorter when ICI 118,551 bound to β_2_AR. We also analyzed the RMSD of the backbone atoms of α5-helix and TM3,5,6,7 (see Figure S3). It could be seen that both of the studied systems reached equilibrium in 200 ns. The β_2_AR-ICI 118,551 system had larger RMSD value of α5-helix and TM3,5,6,7 than the β_2_AR bound to alprenolol and BI-167107. It also suggested that the conformation of α5-helix and TM3,5,6,7 had a larger structural fluctuation when ICI 118,551 combined with β_2_AR. Besides, we also calculated the centroid distance of Gαs and Gβγ domain (Figure 8B). The centroid distance of Gαs and Gβγ domain kept in about 37 Å when alprenolol, BI-167107 or no ligand bound to β_2_AR. In contrast, Gαs and Gβγ domain was separated obviously from each other after 43 ns MD simulations when ICI 118,551 bound to the pocket of β_2_AR. Movie S1 gave a detailed animation about the separation or association of Gαs and Gβγ induced by different ligands. This dissociation was almost accompanied with the relative movement of α5-helix. When the relative motion of α5-helix stopped at about 43 ns, the Gαs and Gβγ were separated from each other (see Figure 8A and 8B). At the same time, we could see the RMSD of β_2_AR changed after about 26 ns (Figure 5A). After another 17 ns, Gαs moved away from Gβγ. It suggested the inverse agonist ICI 118,551 induced the separation of Gαs and Gβγ though changing the conformation of β_2_AR.

The above results indicated that different kinds of ligands could induce the different behaviors of Gαs and Gβγ through changing the conformation of β_2_AR. The Gαs and Gβγ domain were not stable when ICI 118,551 bound to β_2_AR. In contrast, Gαs and Gβγ domain would keep the stable distance if BI-167107, alprenolol or no ligand bound to β_2_AR [9].

### Conclusions

In this study, we focused on the binding mode between β_2_AR and different ligands and the conformational states of β_2_AR in complex with Gαs and Gβγ domain. The hydrogen bonds occupancy showed that Alprenolol, BI-167107 and ICI 118,551 in the pocket of β_2_AR formed different number of hydrogen bonds with the binding site of β_2_AR. These different binding modes would affect the pocket volume size of β_2_AR. The changes of pocket space further affected the conformation of β_2_AR. The results of RMSD indicated that ICI 118,551 could induce β_2_AR to change from active conformation to inactive state. The other ligands were inclined to keep β_2_AR active. Specially, the energy landscape showed three main deep wells when the ICI 118,551 bound with β_2_AR. It suggested ICI 118,55 could induced the conformational change of Gαs and Gβγ. The analysis of IED and centroid distance further illustrated the inactive conformation of β_2_AR induced by ICI 118,551 could lead to the dissociation of Gαs and Gβγ. In comparison, the Gαs and Gβγ would maintain the relative stable distance if there was alprenolol, BI-167107 or no ligand in the active site of β_2_AR (Figure 8C). In total, our MD simulations and energy landscape results demonstrated that different ligands-bound β_2_AR induced the dissociation of downstream Gαs and Gβγ. These results not only depicted the detail dissociation mechanism of Gαs and Gβγ domain which was adjusted indirectly by different ligands, but also could give more clues for the design of potential ligands with different modulating functions.

## Materials and Methods

### Protein Structures Preparation

The agonist-bound model of β_2_AR was prepared beginning from the crystal structure (PDB ID: 3SN6) [12] by removing T4 lysozyme and nanobody (Nb35). Because TM5 and TM6 played an important role in the interaction between β_2_AR and Gs, the missing intracellular loop 3 was added by using the loop model algorithm of MODELLER [38] (see Protocol S1). The neutral antagonist (alprenolol) was extracted from the model (PDB ID: 3NYA) [39]. The inverse agonist (ICI 118,551) was obtained from the crystal structure (PDB ID: 3NY8) [39]. In order to obtain the protein-ligand complex, the inverse agonist and neutral antagonist were docked into the pocket of β_2_AR using AutoDock Vina program [40]. The docking complexes were then used as the starting models for membrane location. The model of β_2_AR-Gs was embedded into an explicit 1-palmitoyl-2-oleoyl-sn-glycero-3-phosphocholine (POPC) by using VMD program [36]. The orientation of membrane was described in Protocol S1 and Figure 1. The length and width of lipid box was 120 Å × 120 Å. The TIP3P water model [41] was used to build the water box which dimensions were 120 Å × 120 Å × 150 Å. Seven sodium ions were added to neutralize the system which contained about 200,010 atoms per periodic cell. The CHARMM force field parameterizations of BI-167107, alprenolol and ICI 118,551 were developed by using VMD Paratool Plugin v1.2 [42] and Gaussian 98 Revision A.9 [43]: The RHF/6–31G* model was used with tight SCF convergence criteria for geometry optimization calculation. The single point calculation was computed at the theory of RHF/6–31G* level with tight SCF convergence criteria.

### Molecular Dynamics Simulations

The β_2_AR-Gs in complex with alprenolol, BI-167107, ICI 118,551 or without ligand were built with explicit lipids and water, respectively. In order to equilibrate these four systems, firstly, each system was fixed except lipid tail for minimizing 100 ps and equilibrating 1000 ps under constant temperature (300 K) and constant pressure (1 bar). Secondly, each system was minimized for 500 ps and equilibrated for 0.5 ns with protein and ligand constrained. Then, 5 ns equilibrated simulations were performed without any constraint. At last, a total of 200 ns MD simulations were performed on the each system under a constant temperature of 300 k and a constant pressure of 1 bar.

Our MD simulations were performed with time step of 2 fs in explicit water and periodically infinite lipid through using NAMD package (version 2.9b3) [44] with CHARMM27 force field [45]. The minimization was based on a conjugate gradient method. The particle-mesh Ewald (PME) [46] method was used to calculate electrostatics with a 12 Å nonbonded cutoff. Langevin piston and Langevin barostat methods were employed for the temperature and pressure respectively [47]. The frames were saved every 20.0 ps during the MD simulations.

All MD simulations were performed on the GPU workstation. In order to get the highest efficiency of GPU, the speed test of GPU workstation was carried out with different collocations of Cores and GPU (see Figure S1).The speed test results proved that running on 12 cores of an array of two 2.66-GHz Intel Xeon 5650 processors and 4 pieces of NVDIA Tesla C 2050 graphics card could get the highest speed. The wall clock time was about 3.46 ns per day.

### Hydrogen Bonds and Volume Calculation

In the statistical analysis of the hydrogen bonds occupancy, the distance and angle between the acceptor and donor atoms were set less than 3.5 Å and 35°, respectively [48], [49]. The polyhedral volumetric model of the pocket detection plugin of VMD [35], [36] was used to find the pocket volume of β_2_AR.

### Interactive Essential Dynamics Analysis

For the interactive essential dynamics (IED) analysis [33], the complex were split into three parts: β_2_AR, Gαs and Gβγ. 25 eigenvectors were generated for each part on the basis of trajectory file, then 25 projections were obtained from eigenvectors. The IED was calculated by equation 1:
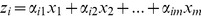
(1)


Where 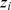 represented the *i*th principal component. 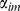
was weight coefficient. 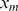 represented the position. The first two components could represent the main motions of protein. More details about IED method were described in the Text S1. Trajectory analysis was carried out using AmberTools12 and VMD [36], [50].

### Energy Landscape Analysis

The energy landscape of the conformational change of protein complex could be estimated by an appropriate conformation sampling method. In order to get the a two dimensional (2D) energy landscape map, the centroid distance between Gαs and Gβγ, which mainly represented the motion, and the RMSD of Gαs and Gβγ, which corresponded the conformational fluctuation, were chosen as the reaction coordinates. The energy landscape could be calculated along these two reaction coordinates as equation 2 [51]–[54] shown:
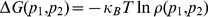
(2)


Where 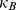 represented the Boltzmann constant, T was the simulated temperature, and 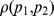 represented the normalized joint probability distribution.

## Supporting Information

Figure S1 **Speed test of GPU workstation.** Workstation with 12 Cores+4GPU gives the fastest speed.
(TIF)

Figure S2 **RMSD of backbone atoms of β_2_AR versus 5 ns MD simulations time.**
(TIF)

Figure S3 **Time evolution of RMSD of the backbone atoms of α5-helix and TM 3,5,6,7.**
(TIF)

Table S1 **RMSD of simulated conformational backbone atoms with respect to the crystal structure of ICI 118,551-bound β_2_AR.**
(DOC)

Text S1 **Interactive Essential Dynamics.**
(DOC)

Protocol S1 **Membrane building protocol.**
(DOC)

Movie S1 **Animation about the separation or association of Gαs and Gβγ induced by different ligands.**
(AVI)
