# Supplementary material for: Computational Study on the Different Ligands Induced Conformation Change of β2 Adrenergic Receptor-Gs Protein Complex
Source: PLoS One. 2013 Jul 29;8(7):e68138. doi: 10.1371/journal.pone.0068138 (PMC3726664; doi:10.1371/journal.pone.0068138)
Supplement: Table S1 — RMSD of simulated conformational backbone atoms with respect to the crystal structure of ICI 118,551-bound β2AR. (DOC) [file pone.0068138.s004.doc]

***Table S1***

**Table S1.** RMSD of simulated conformational backbone atoms with respect to the crystal structure of ICI 118,551-bound β_2_AR.

| Time (ns) | RMSD ^a^ (Å) |
| --- | --- |
| 0 | 3.74 |
| 50 | 2.11 |
| 100 | 1.72 |
| 150 | 1.68 |
| 200 | 1.76 |

^a^RMSD based on the backbone of the inverse agonist-bound β_2_AR (PDB code: 3NY8)
