# Supplementary material for: Computational Study on the Different Ligands Induced Conformation Change of β2 Adrenergic Receptor-Gs Protein Complex
Source: PLoS One. 2013 Jul 29;8(7):e68138. doi: 10.1371/journal.pone.0068138 (PMC3726664; doi:10.1371/journal.pone.0068138)
Supplement: Text S1 — Interactive Essential Dynamics. (DOC) [file pone.0068138.s005.doc]

***Text S1***

**Interactive Essential Dynamics**

Interactive essential dynamics (IED), often called principal component analysis of molecular dynamics trajectory data, was frequently used to elucidate the domain motion of large molecules such as protein, nucleic acid, *etc*. The procedure of IED was described as below:

Firstly, the set of atoms should be set an index of selectivity for analysis. The index of selectivity could be all atoms, all non-hydrogen atoms, all alpha carbons or subset atoms according to different situations. When all atoms were chosen in the analysis, IED could discover the large scale global motion. If the localized motions need to be analyzed, the subset of atoms should be selected.

Secondly, the rotation and translation of molecule should be removed because they did not contribute the real dynamics of the system. The root mean square deviation (RMSD) was calculated for the alignment to fit on the selected atoms as shown in equation (1).

(1)

Where $x_{n}$ and $y_{n}$ were two given the vector sets of atom coordinates and $W_{n}$ was the weights. $U$ was all possible orthogonal matrices. The best fit and rotation were that E has minimum value when $U$ took a certain value.

Once the selected atoms had been aligned, the covariance matrix should be computed. The average position of each atom was measured along the entire MD trajectory. The calculation of two set atoms was shown in equation (2). If each of two set atoms were largely along the same deviation direction of their equilibrium positions, the covariance matrix element of those two set atom would be positive and large.

(2)

Where $\langle x_{i}\rangle$ and $\langle y_{j}\rangle$ represented the average position and $c_{ij}$ was the covariance matrix element.

At last, the principal component was defined as shown in equation (3). A much smaller number of principal components could account for important data variance in proteins and nucleic acids. For instance, three principal components might contain 85% of the variance information in the case of nucleic acids. So important motions of proteins or nucleic acids could be extracted from the first two or three principal components.

(3)

Where represented the *i*th principal component. was weight coefficient. $x_{i}$ represented the position.
