# Supplementary material for: Computational Study on the Different Ligands Induced Conformation Change of β2 Adrenergic Receptor-Gs Protein Complex
Source: PLoS One. 2013 Jul 29;8(7):e68138. doi: 10.1371/journal.pone.0068138 (PMC3726664; doi:10.1371/journal.pone.0068138)
Supplement: Protocol S1 — Membrane building protocol. (DOC) [file pone.0068138.s006.doc]

***Protocol S1***

**Membrane building protocol**

Firstly, the missing atoms between TM5 and TM6 were added using MODELLER software. The sequence of the missing amino acids was as below:

(FHVQNLSQVEQDGRTGHGLRRSSKF).

The alignment algorithm of MODELLER program was used to build new sequence on basis sequence of crystal structure of β_2_AR-Gs, as well as using to optimize missing atoms part.

Secondly, when the structure of β_2_AR-Gs was built, the complex were solvated by using SOLVATE program (*http://www.mpibpc.mpg.de/272144/07-solvate*). The water layer thickness with 3 Å was used.

At last, the orientation of the protein in the membrane could be discovered though the hydrophobicity of its residues. In order to find the membrane location in a more rigorous way, the protein was submitted into PPM Server. The depth hydrophobic thickness was 30.0±1.0 Å as shown in Figure 1. The membrane embedded 799 residues containing TM1 (31-56), TM2 (71-92), TM3 (104-129), TM4 (151-170), TM5 (198-220), TM6 (275-295), TM7 (306-326). At the same time, water molecules were eliminated from the hydrophobic protein membrane.
